# The electrospinning of the copolymer of styrene and butyl acrylate for its application as oil absorbent

**DOI:** 10.1186/s40064-016-2845-6

**Published:** 2016-08-22

**Authors:** Naiku Xu, Jipeng Cao, Yuyao Lu

**Affiliations:** 1 State Key Laboratory of Separation Membranes and Membrane Processes, School of Material Science and Engineering, Tianjin Polytechnic University, Tianjin, 300387 China; 2 Liaoning Key Laboratory of Functional Textile Materials, School of Clothing & Textiles, Eastern Liaoning University, Dandong, 118003 China

**Keywords:** Synthesis, Poly (styrene-co-butyl acrylate), Electrospinning, Fibrous membrane, Oil absorbent

## Abstract

Electrospun polystyrene materials have been employed as oil absorbents, but they have visible drawbacks such as poor strength at low temperature and unreliable integrity because of brittleness and insufficient cohesive force among fibers. Butyl acrylate can polymerize into flexible chains, and its polymer can be used as elastomer and adhesive material. Thereby it is possible to obtain the material that has better performance in comparison with electrospun polystyrene material through the electrospinning of the copolymer of styrene and butyl acrylate. In this work, a polymer was synthesized through suspension polymerization by using styrene and butyl acrylate as comonomers. The synthesis of the copolymer of styrene and butyl acrylate was verified through dissolution and hydrolysis experimental data; as well through nuclear magnetic resonance spectrometry. The viscous flow activation energy of the solution consisting of copolymer and N, N-dimethylformamide was determined via viscosity method and then adopted to establish the entanglement characteristics of butyl acrylate’s chain segments. Finally, in order to electrospin the copolymer solution into fibrous membrane, the effects of monomer feed ratio and spinning parameters were investigated. The prepared fibrous membrane was found to have a potential use as oil absorbent.

## Background

Due to the thriving development of industry around the world, oily organic liquids have been manufactured massively and caused water pollution. These liquids are usually toxic and insoluble in water, thus they spread on water surface to cause severe damage for ecological environment and human being’s health (Nriagu et al. 2016; Adamu et al. 2015; Wan et al. 2014). As a result, it is an urgent task for mankind to control the pollution in time. In addition, it is also important to reclaim valuable organic liquids from the polluted water.

In order to separate oily organic liquids from the polluted water, oil absorbents have been used widely. Wu et al. (2014) prepared polyurethane sponge via surface modification, and they found that the sponge had an oil absorption capacity of more than 100 g/g. Zhang et al. (2015) chose carbonyl iron powders as magnetic material to prepare magnetic poly(styrene–divinylbenzene) monoliths with porous structure and lipophilicity through direct molding and controlled polymerization, and they found that the monoliths had an oil intake capacity of approximately 23 times its own weight. Yati et al. (2016) prepared an oil absorbent with a cross-linked 3-dimensional network via the condensation of poly(tetrahydrofuran) with tris[3-(trimethoxysilyl)propyl] isocyanurate, and they found that the absorbent had a high and fast swelling capacity in various oils. Up to now, some achievements have been made in the field of absorbents preparation. However, granular absorbents can induce a secondary pollution since they can hardly be collected from water after application; spongeous or monolithic absorbents can be collected from water after application, but they exhibit relatively low specific surface area, thus the application values of these absorbents have been shrunk greatly.

Fibrous materials are well-known for large specific surface area, fast absorption rate, and excellent actual operability. Some researchers have reported a lot of fibrous materials which can be used as oil absorbents, for example, the fibers prepared via dry-wet spinning (Feng and Xiao 2006), gelation spinning (Xu and Xiao 2010), wet spinning (Zhao et al. 2011), melt spinning (Zhao et al. 2012), reactive extrusion-melt spinning (Ma et al. 2013), and graft modification (Li and Wei 2012). Due to good practicability and wide applicability, non-woven fabrics, such as melt blown polypropylene non-woven fabric (Zhao et al. 2013a, b), are another important fibrous material which can be used as oil absorbent (Radetic et al. 2008). The above-mentioned fibrous materials involving the fibers and non-woven fabrics can absorb a large amount of oily organic liquids; however, since the fibrous materials are composed of thick fibers, they absorb organic liquids at a relatively slow rate, which has an adverse effect on their application. Electrospun fibrous materials are of great advantage to oil absorption rate for the reason that the diameter of electrospun fibers can reach nanometer level (Zhu et al. 2011). In recent years, some polymers, such as polyacrylonitrile (Su et al. 2012; Liu et al. 2014), poly(m-phenylene isophthalamide) (Tang et al. 2013), and cellulose acetate (Shang et al. 2012), have been electrospun into fibrous materials and used as oil absorbents to separate oil from water. Polystyrene, as a commonly solvent-soluble and thermoplastic polymer, has more excellent electrospinnability in comparison with other polymers, thus electrospun polystyrene fibrous material has been used widely as oil absorbent (Lin et al. 2012, 2013; Lee et al. 2013; Wu et al. 2012). However, polystyrene is a kind of rigid polymer, thus its electrospun fibrous material exhibits some disadvantages. First, some fibers can fall off the fibrous material due to weak cohesive force among fibers. Second, owning to low temperature brittleness, some fibers break at low temperature, and the fibrous material shows poor strength. Therefore, it is very necessary to improve the performance of electrospun polystyrene fibrous material. The previous work showed that electrospun poly (meth)acrylate fibers could adhere to each other to provide a strong cohesive force for fibrous material (Mo et al. 2014); additionally, poly (butyl acrylate) has good low temperature resistance because it has the properties similar to those of natural rubber. Thereby it was proposed that to copolymerize with butyl acrylate was an effective approach to supply electrospun polystyrene material with good cohesive force and flexibility. As a consequence, the shortages of electrospun polystyrene material could be remedied easily.

In this study, suspension polymerization was first adopted to synthesize a polymer of styrene and butyl acrylate in the presence of benzoyl peroxide and polyvinyl alcohol as initiator and stabilizing agent. Dissolution and hydrolysis means, along with nuclear magnetic resonance (NMR) spectrometry, were used to prove the successful copolymerization of styrene with butyl acrylate. Thereafter, the viscous flow activation energy determined via viscosity test was used to confirm the entanglement characteristics of butyl acrylate’s chain segments. Finally, field emission scanning electron microscopy (FESEM) was employed to analyze the effect of spinning parameters on morphological structure of fibrous membrane. Oil absorption capacities of the fibrous membranes of polystyrene and poly (styrene-co-butyl acrylate) were compared preliminarily, and morphological difference between the fibrous membranes of polystyrene and poly (styrene-co-butyl acrylate) was used to explain the difference of oil absorption. In addition, the mechanism of oil absorption was proposed.

## Experimental

### Materials

Butyl acrylate (BA) was provided by Tianjin Guangfu Fine Chemical Research Institute. Styrene (St) was purchased from Tianjin Fuchen Chemical Reagents Factory. Benzoyl peroxide (BPO) was purchased from China National Pharmaceutical Group Corp. Shanghai Chemical Reagent Co., Ltd. N, N-dimethylformamide (DMF) was supplied by Tianjin Guangfu Science and Technology Development Co., Ltd. Motor oil was provided by Shell Tongyi (Beijing) Petroleum Chemical Co., Ltd. Soybean oil was provided by Kerry Oils & Grains (Tianjin) Co., Ltd. Pump oil was provided by Tianjin Shifang Chemical Co. Ltd. Isopropanol was provided by Tianjin Fengchuan Chemical Reagent Co. Ltd. The above-mentioned materials were used as-received. Polyvinyl alcohol (PVA) was supplied by Hunan Xiangwei Co., Ltd. and used after water washing and drying in a vacuum oven.

### Copolymerization

A solution was first prepared by dissolving 2.25 g PVA in 100 ml deionized water at 80 °C, and the solution was then cooled down to room temperature. Another solution was subsequently obtained by mixing BPO whose mass was equal to 0.5 % of the total mass of St and BA with the mixture of St and BA at room temperature. The mass fraction of BA in the mixture of St and BA with a total volume of 150 ml was assigned to 0, 20, 30, or 40 %. Thereafter, the two solutions and 350 ml deionized water were stirred to react at 85 °C for 3 h and 95 °C for 3 h in a three-neck flask under nitrogen atmosphere. After being separated from the solution of PVA and deionized water via vacuum filtering, the resultants were washed initially with hot water and subsequently with deionized water. Finally, four kinds of polymers synthesized when the mass fraction of BA was equal to 40, 30, 20, or 0 % were labeled as 1#, 2#, 3#, and 4# after drying in a vacuum oven at 33 °C.

### Homopolymerization

A solution was first prepared by dissolving 2.25 g PVA in 100 ml deionized water at 80 °C, and the solution was then cooled down to room temperature. Another solution was subsequently obtained by mixing BPO whose mass was equal to 0.5 % of the mass of BA with 150 ml BA at room temperature. Thereafter, the two solutions and 350 ml deionized water were stirred to react at 85 °C for 5 h and 95 °C for 1 h in a three-neck flask under nitrogen atmosphere. After being separated from the solution of PVA and deionized water via vacuum filtering, the resultants were washed initially with hot water and subsequently with deionized water. Finally, poly (butyl acrylate) (PBA), labeling as 5#, was obtained through drying in a vacuum oven at 33 °C.

### NMR analysis

An Avance 300 MHz (Bruker Corp., Germany) nuclear magnetic resonance (NMR) spectrometer was used to analyze the macromolecular structure of the above-synthesized polymer. The test was performed at resonance frequencies of 300 and 75 Hz for ^1^H and ^13^C nuclei at 20 °C. The spectra were obtained by using deuterated chloroform (CDCl_3_) as solvent, and chemical shifts were referenced to tetramethylsilane (TMS).

### Dissolution

The above-synthesized polymer with a known mass was put into a small bag sewed by polypropylene nonwoven fabric. After weighing total mass, the bag was immersed in DMF for 1 h at 90 °C. After being separated from DMF and dried in a vacuum oven at 75 °C, the bag that contained residual polymer was weighed. The remaining ratio of polymer after dissolution by DMF, R (%), was calculated according to the following equation:

1 $$ {\text{R}} = 100\;\% - \left( {{\text{G}}_{\text{a}} - {\text{G}}_{\text{b}} } \right)/{\text{G}}_{ 0} \times 100\;\% $$

where G_a_ is the total mass of dried polymer and bag before dissolution (g); G_b_ is the total mass of dried polymer and bag after dissolution (g); G_0_ is the mass of dried polymer before dissolution (g).

### Hydrolysis

30 g isopropanol was first used as swelling agent to swell the above-synthesized polymer with a mass of 0.5 g in a beaker at 40 °C for 6 h, and the swollen polymer was then separated from isopropanol through filtration. Thereafter, the polymer was hydrolyzed in 30 g aqueous solution of sodium hydroxide with a mass concentration of 50 % at 90 °C for 6 h. After being separated from the lye, the hydrolyzed polymer was washed by boiling deionized water and dried in a vacuum oven at 75 °C for 24 h. The remaining ratio of polymer after hydrolysis by NaOH, H (%), was calculated according to the following equation:

2 $$ {\text{H}} = {\text{G}}_{\text{H}} /{\text{G}}_{\text{pre}} \times 100\;\% $$

where G_pre_ is the mass of polymer before swelling and hydrolysis (g); G_H_ is the mass of dried polymer after hydrolysis (g).

### Spinning solution preparation

The above-synthesized polymer with a mass of 3 g was mixed with 17 g DMF in a beaker, and the mixture was then stirred at 80 °C until the polymer completely dissolved. The solution was preserved with a dry and clean beaker for later use.

### Viscosity test

The spinning solutions of the polymer 1# or 4# were used for analysis, their viscosities were measured by using a SNB-1A rotary digital viscometer (Shanghai Fangrui Instrument Co., Ltd., China). The test was performed at 30, 40, 50, 60, and 70 °C when the rotor 21# rotated with a speed of 20 r/min.

### Fibrous membrane preparation

**Fig. 1:**
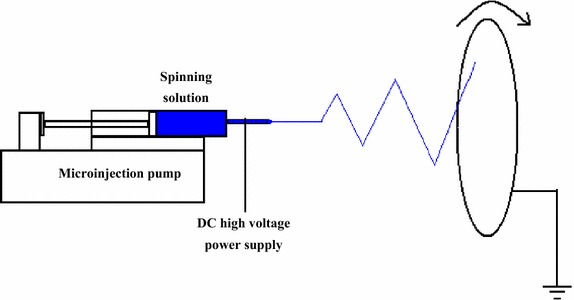
A diagram of electrospinning apparatus

As shown in Fig. 1, the spinning solution was sucked into a 10 ml plastic syringe, and the syringe was subsequently connected with a metallic needle whose inner diameter was equal to 0.6 mm. The syringe was horizontally fixed on a microinjection pump which was used to provide a proper extrusion rate for the spinning solution. A DC high voltage power supply was in contact with the needle to provide a high voltage, and a grounded collector was positioned at the distance of 20 cm away from the needle tip. The electrospun fibers were received by the grounded collector covered by aluminum foil to form fibrous membrane. The spinning solution was extruded from the needle at a rate within the range of 0.4–1.2 ml/h while a high voltage within the range of 12–18 kV was applied. In addition, the grounded collector rotated at a speed of 200 r/min.

### Morphology observation

The surface of fibrous membrane was first coated with gold via an electro-deposition method, and its surface morphology was then observed by a NOVA NANOSEM 230 (FEI, America) field emission scanning electron microscopy (FESEM) at an accelerating voltage of 15.0 kV.

### Oil absorption capacity test

Soybean oil, pump oil, and motor oil were selected as oily organic liquids, and the fibrous membrane with a known mass was in contact with the organic liquids for 180 min. Then, the fibrous membrane containing absorbed organic liquid was weighed. The organic liquid absorbency, OLA (g/g), was calculated according to the following equation:

3 $$ {\text{OLA}} = \left( {{\text{G}}_{ 1} - {\text{G}}_{ 0} } \right)/{\text{G}}_{0} $$

where G_1_ is the total mass of fibrous membrane and absorbed organic liquid; G_0_ is the initial mass of fibrous membrane.

## Results and discussion

### Macromolecular structure

**Fig. 2:**
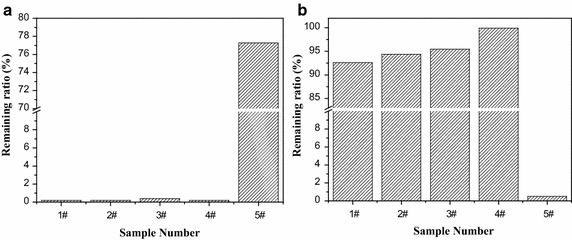
Remaining ratios of the synthesized polymers after dissolution by DMF (**a**) and hydrolysis by NaOH (**b**)

BA is easy to polymerize into a branched polymer by these modes including intramolecular bite-backing, intermolecular hydrogen capture, and β cracking (Yu-Su et al. 2011). As shown in Fig. 2a, after dissolution by DMF, the polymer 5# had a remaining ratio of about 78 %. This result meant that branching process occurred during the suspension homopolymerization of BA, the branched chains made the synthesized PBA slightly soluble in DMF. For the suspension polymerization of BA and St, if BA only conducted a homopolymerization reaction, the generated polymer would have a remaining ratio of much higher than 0 % due to the presence of branched chains. In fact, the polymer generated from the suspension polymerization of BA and St has a remaining ratio of around 0 %, as shown in Fig. 2a, thus BA should conduct a copolymerization reaction. In addition, after hydrolysis by NaOH, the polymer 5# had a remaining ratio of about 0 % while the polymer 4# had a remaining ratio of about 100 %, as shown in Fig. 2b. This result indicated that PBA could hydrolyze to turn into water-soluble polymer, but the lye could not change polystyrene’s solubility in water. In this case, if the polymers 1#, 2#, and 3# only consisted of BA homopolymer and St homopolymer, after hydrolysis by NaOH, their remaining ratios would be higher than 60 % and less than 80 % in that the total yield of each polymerization reached 99 % and the mass ratio of BA to St in monomer feed was within the range of 2:8–4:6. In practice, the polymer 1#, 2#, or 3# had a remaining ratio of higher than 93 %, as shown in Fig. 2b, thus poly (styrene-co-butyl acrylate) was the main component for the polymers 1#, 2#, and 3#.

**Fig. 3:**
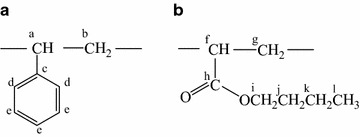
Structural unit formulae of styrene (**a**) and butyl acrylate (**b**)

**Fig. 4:**
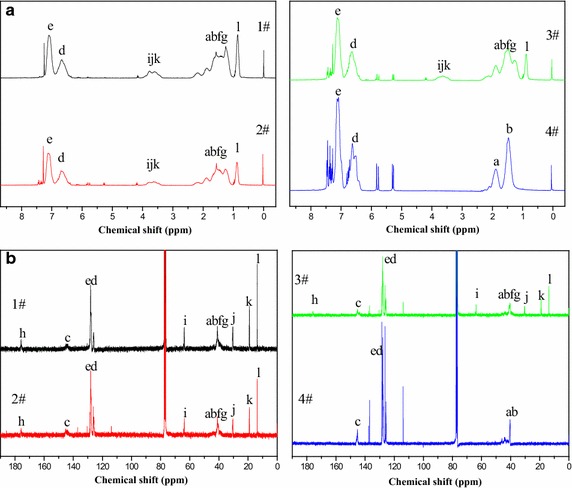
^1^H-NMR spectra (**a**) and ^13^C-NMR spectra (**b**) of the synthesized polymers 1#, 2#, 3#, and 4#

The above conclusion was also proved by ^1^H and ^13^C NMR spectra, as shown in Figs. 3 and 4. For the polymer 4#, the peaks within the band of 7.3–6.7 ppm were the characteristic peaks of phenyl’s protons (e, d) (Verebelyi et al. 2012). The signals of phenyl’s protons of 3#, 2#, and 1# became weaker in comparison with the one of 4#, indicating that the phenyls of 3#, 2#, and 1# had a chemical environment different from the one of 4#. The protons of CH_2_ and CH (b, a) made the polymer 4# exhibit another two clear peaks at 1.8 and 1.5 ppm (Kowalewska 2008); however, these protons’ chemical environments changed with the introduction of BA, and the peaks of the protons of CH and CH_2_ (f, g) appeared near 1.5 ppm, which was in accordance with the findings of Chiu et al. (2006). There were no peaks within the range of 3.3–4.3 ppm for the polymer 4#. The peaks caused by the protons of CH_2_ (k, j, i) appeared within the band of 3.3–4.3 ppm for the polymers 3#, 2#, and 1# (Gichinga et al. 2010); additionally, the peak caused by the protons of CH_3_ (l) appeared at 0.85 ppm (Wang and Zhang 2012). In addition, two peaks at 128 and 126 ppm caused by phenyl’s carbons (e, d) (Arehart and Matyjaszewski 1999) became weak with the use of BA as comonomer. The peaks at 13.7, 19.0, 30.4, and 63.8 ppm caused by the carbons of CH_3_ and CH_2_ (l, k, j, i) (Wyzgoski et al. 2004) appeared for the polymers 1#, 2#, and 3# but did not appear for the polymer 4#. The peak at 175.9 ppm which was attributed to the characteristic peak of the carbon of COO (h) (Kopchick et al. 2008) also appeared for the polymers 1#, 2#, and 3#, and got more and more obvious from 3# to 1#. Therefore, there was no doubt that St copolymerized with BA to produce poly (styrene-co-butyl acrylate) during suspension polymerization.

### Viscous flow active energy

Eyring et al. reported that the viscous flow activation energy (E_η_) of pure liquid could be calculated via the following equation (Glasstone et al. 1941):

4 $$ \eta = \left( {hN_{A} /V} \right){ \exp }\left( {E_{\eta } /RT} \right) $$

where N_A_ and h are Avogadro’s number and Planck’s constant, respectively, V is molar volume, R is gas constant, and T is thermodynamic temperature.

If a symbol A was used to replace hN_A_/V, the above equation would be transformed into the following linear equation:

5 $$ Ln\eta = LnA + E_{\eta } /RT $$

**Fig. 5:**
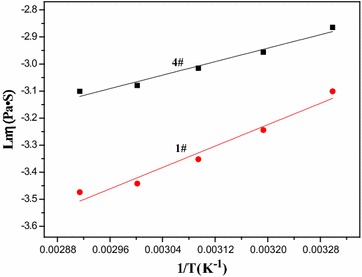
The curves of Lnη versus 1/T for the spinning solutions of the synthesized polymers 1# and 4#

According to Eq. (5), E_η_ could be obtained from the slope of the linear equation of Lnη versus 1/T. The curves of Lnη versus 1/T for the spinning solutions are illustrated in Fig. 5. E_η_ of the spinning solution of the polymer 1# was found to be 8.22 kJ/mol while E_η_ of 4#’s spinning solution was 5.18 kJ/mol. Such result indicated that BA chain segments raised E_η_ of spinning solution. According to Hao and his coworkers’ findings, the viscous flow activation energy of polymer solution depended on macromolecular entanglements in solution (Hao et al. 2007); additionally, BA chain segments were soft and flexible in comparison with St chain segments, thus it was deduced that BA chain segments made poly (styrene-co-butyl acrylate) chains entangle with each other in spinning solution. It is well known that entanglement is highly advantageous for electrospinning since it can provide good cohesive force for electrospun fibers (Gupta et al. 2005). Thereby, poly (styrene-co-butyl acrylate) had better electrospinnability in comparison with polystyrene.

### Morphology difference

**Fig. 6:**
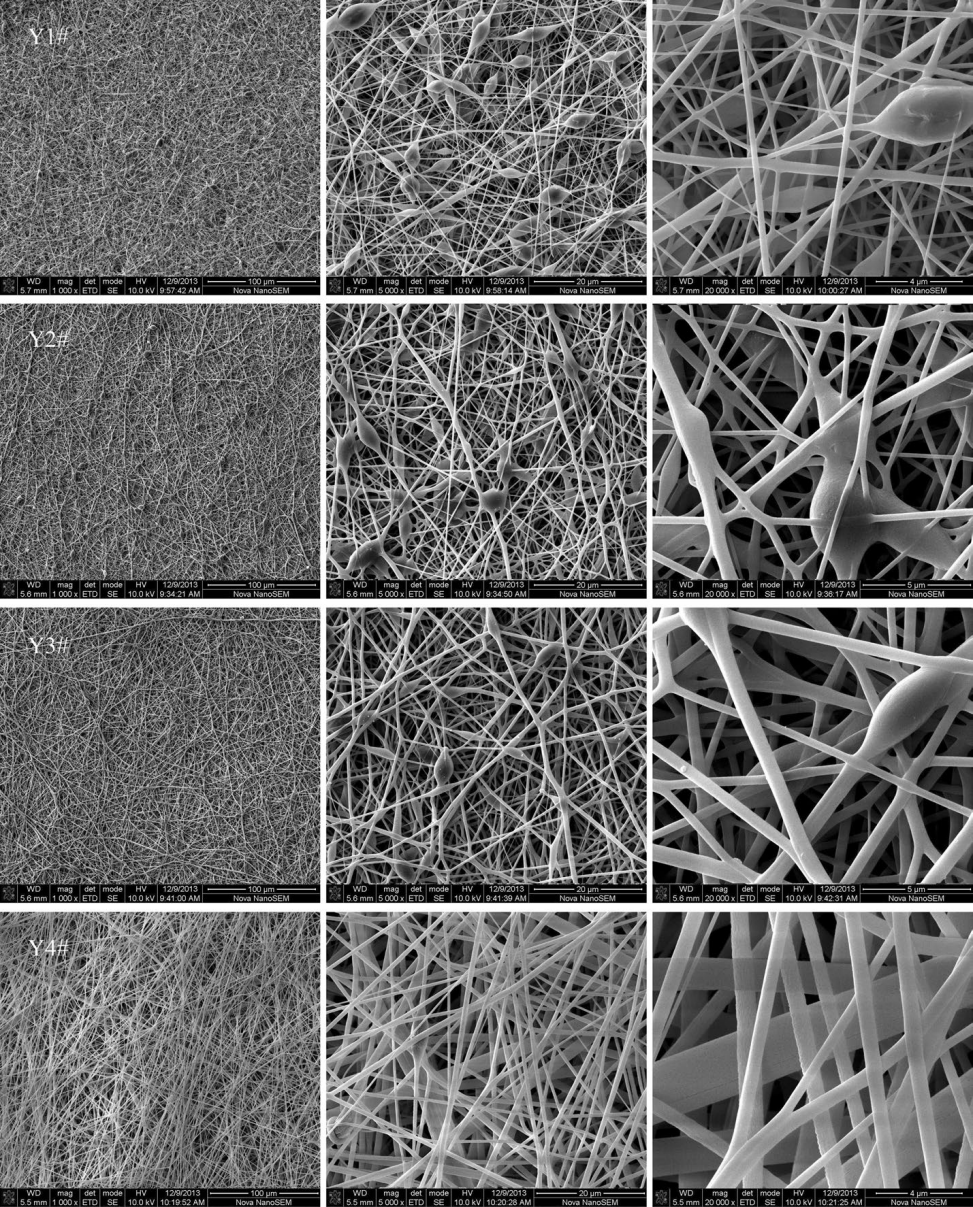
The morphologies of the fibrous membranes Y1#, Y2#, Y3#, and Y4# electrospun from the spinning solutions of the synthesized polymers 1#, 2#, 3#, and 4# at an extrusion rate of 0.8 ml/h and an applied voltage of 15 kV

Figure 6 shows the morphology difference among the fibrous membranes prepared from the polymers 1#, 2#, 3#, and 4#. The fibrous membranes Y1#, Y2#, and Y3# showed the structures of beads-on-a-string, and the number of beads decreased markedly as the mass ratio of BA to St in monomer feed changed from 6:4 to 8:2. The fibrous membrane Y4# did not exhibit a beads-on-a-string structure. According to the findings of Sattler et al. (2012), the beads-on-a-string structure originated from a relaxed state of polymer chains in the earliest stages of pinching; the polymer chains inside a bead remained relaxed while the polymer chains in a string became stretched, thus it was deduced that relaxed and stretched polymer chains coexisted for the fibrous membranes Y1#, Y2#, and Y3#, but only stretched polymer chains existed for the fibrous membrane Y4#. Electrospinning can create extreme elongation and thinning of the order of 10^5^ for the resulting jet, accompanied by a strain rate of the order 10^3^ s^−1^, and the process duration is within 10^−2^ s (Malkin et al. 2014). According to Han et al. (2008), the initial longitudinal stresses relaxed at a distance of about 2–4 cm away from the transition zone that connected the modified Taylor cone and the thin segment, beyond this distance, a high strain rate was imposed on the jet, the relaxation of polymer chains became impossible. Due to fast strain rate, short duration, and short relaxation distance, rigid polystyrene chains could hardly relax during electrospinning, thus the polymer chains in the fibrous membrane Y4# were stretched, resulting in a morphological structure without beads; however, soft and flexible BA chain segments could relax within a short time, consequently, the coexistence of relaxed and stretched polymer chains made the fibrous membranes Y1#, Y2#, and Y3# form the morphological structures of beads-on-a-string.

### Effect of spinning parameters

**Fig. 7:**
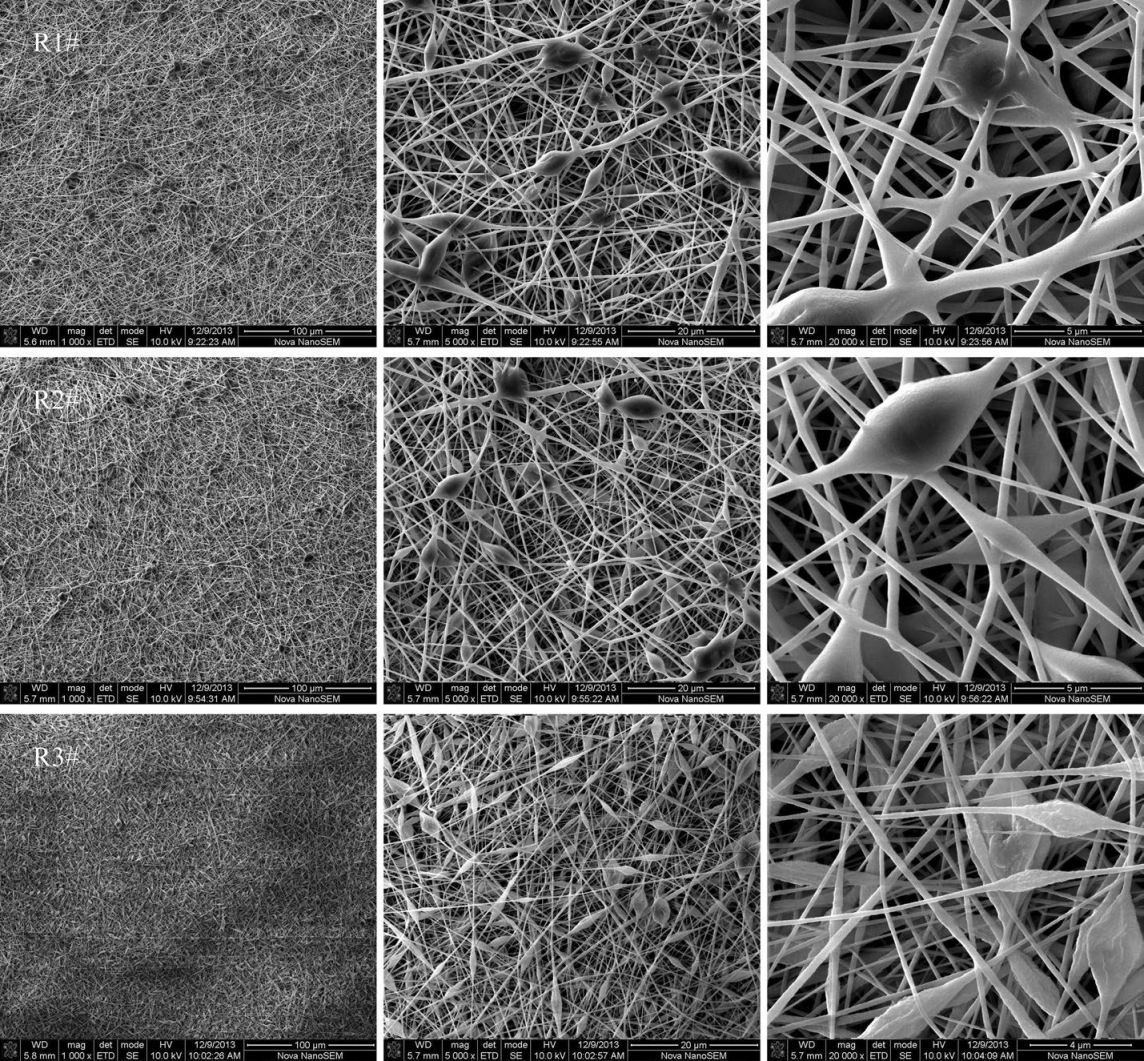
The morphology of the fibrous membrane electrospun from the spinning solution of the synthesized polymer 1# at an applied voltage of 15 kV; R1# prepared at an extrusion rate of 0.4 ml/h; R2# prepared at an extrusion rate of 0.8 ml/h; R3# prepared at an extrusion rate of 1.2 ml/h

**Fig. 8:**
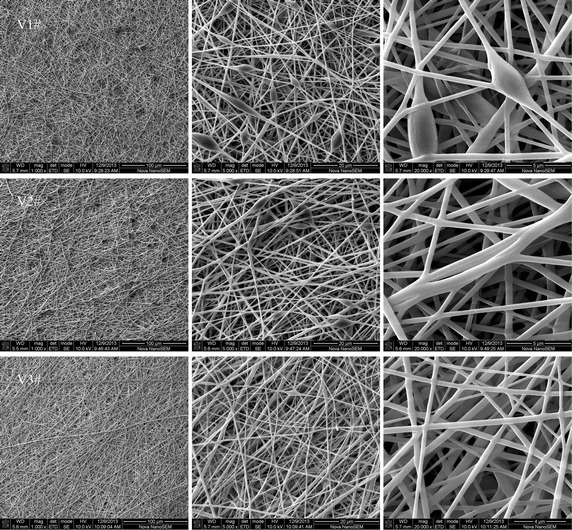
The morphology of the fibrous membrane electrospun from the spinning solution of the synthesized polymer 1# at an extrusion rate of 0.8 ml/h; V1# prepared at an applied voltage of 12 kV; V2# prepared at an applied voltage of 15 kV; V3# prepared at an applied voltage of 18 kV

For the spinning solution with a given concentration, strain rate and fiber formation duration during electrospinning are related to extrusion rate and applied voltage, the effects of extrusion rate and applied voltage on the morphological structure of ectrospun fibrous membrane are shown in Figs. 7 and 8. More beads appeared with increasing extrusion rate from 0.4 to 1.2 ml/h, and the fibrous membrane R3# exhibited a much more obvious structure of beads-on-a-string in comparison with R1# and R2#, as shown in Fig. 7. However, the number of beads decreased obviously with an increase in applied voltage, and the beads disappeared from the fibrous membrane V3#, as shown in Fig. 8. These results suggested that a quick extrusion rate could prompt the relaxation of polymer chains, and a high applied voltage could restrain the relaxation of polymer chains. In comparison with the extrusion rate of 0.4 ml/h, the extrusion rate of 1.2 ml/h could make more solution enter electrostatic field within a given time, and charge amount in the solution decreased when a constant voltage was applied. In this case, the solution moved in the electrostatic field at a slow speed to result in long fiber formation duration; additionally, the decrease of electrostatic repulsion which was also caused by the decrease of charge amount leaded to slow strain rate. Thanks to the slow strain rate and long fiber formation duration, polymer chains had enough time to relax before formation, thus the morphological structure of beads-on-a-string became more obvious with an increase in extrusion rate. On the contrary, the increase of applied voltage could increase the solution’s charge amount, thus the strain rate during electrospinning increased markedly, and the fiber formation duration shortened. Because of the fast strain rate and short fiber formation duration, polymer chains could not relax before formation. As a result, the beads disappeared from the fibrous membrane prepared at a high applied voltage.

### Oil absorption property

**Fig. 9:**
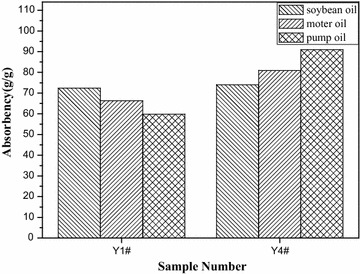
The comparison between oil absorbencies of the fibrous membranes Y1# and Y4#

The fibrous membranes Y1#, Y2#, and Y3# exhibited similar morphological structures while the fibrous membrane Y4# owned a unique morphological structure, thus the fibrous membranes Y1# and Y4# were used as tested samples to reveal the relationship between morphological structure and oil absorption property. The fibrous membrane Y4# had a loose and porous structure, as shown in Fig. 6; however, the fibrous membrane Y1# showed a relatively compact structure. In this instance, the fibrous membrane Y4# would have higher oil absorbency than the fibrous membrane Y1#. As expected, when the oils with high viscosity such as pump oil (167 mPa s determined in the SNB-1A rotary digital viscometer at 25 °C by using 21# rotor at a rotational speed of 10 r/min) and motor oil (224 mPa s determined as the procedures of pump oil) were used as tested liquids, the fibrous membrane Y4#’s absorbency was much higher than that of the fibrous membrane Y1#, as shown in Fig. 9. However, for the oils with low viscosity such as soybean oil (80 mPa s determined as the procedures of pump oil), the fibrous membrane Y4#’s absorbency was close to that of the fibrous membrane Y1#. This reslut implied that loose and porous structure was highly advantageous for the entry and retention of high viscosity oils, but was highly disadvantageous for the retention of low viscosity oils. Therefore, the fibrous membranes electrospun from the synthesized polymers showed good potential application for the absorption of the oils with different viscosities. In fact, the mechanism by which the fibrous membrane absorbed oils should be explained as follows. The fibrous membrane had a network structure that was constructed by numerous superfine fibers, and there were a lot of micro- and nano-pores in the network, as shown in Fig. 6; additionally, since the polymer synthesized above was an oleophilic substance, the surface of the fibrous membrane exhibited very good affinity for oils. Due to the above-mentioned characteristics, when the fibrous membrane was immersed in oils, oils first wetted its surface and then either went into its pores or further swelled the superfine fibers’ surfaces. The pores and swelling region had a strong inhibition for oils escape, thus the fibrous membrane exhibited the capability to absorb oils.

## Conclusions

Styrene reacted with butyl acrylate during suspension polymerization to form a copolymer, poly (styrene-co-butyl acrylate). Soft and flexible butyl acrylate chain segments prompted poly (styrene-co-butyl acrylate) chains to entangle with each other in spinning solution. Rigid polystyrene chains did not relax during electrospinning so that its fibrous membrane owned a morphological structure without beads; however, poly (styrene-co-butyl acrylate) chains relaxed at a faster rate to result in that the fibrous membrane fabricated from poly (styrene-co-butyl acrylate) had a morphological structure of beads-on-a-string. Extrusion rate and applied voltage imposed a great impact on the relaxation of polymer chains through affecting strain rate and fiber formation duration. The fibrous membranes prepared at different extrusion rates or applied voltages showed different morphological structures. Due to the different morphological structures, the fibrous membrane electrospun from poly (styrene-co-butyl acrylate) had a potential use as absorbent for low viscosity oils while electrospun polystyrene fibrous membrane could be used as absorbent for high viscosity oils.
